# From the ICU Bedside: Applying the Transnational Clinical Academic Doctorate Lens to a Clinically Embedded PhD Journey

**DOI:** 10.1111/jan.70421

**Published:** 2025-12-07

**Authors:** Gideon U. Johnson

**Affiliations:** ^1^ Florence Nightingale Faculty of Nursing, Midwifery & Palliative Care King's College London London UK; ^2^ School of Nursing and Midwifery Edith Cowan University Perth Australia

**Keywords:** analytic autoethnography, clinical academic pathway, delirium, doctoral education, ICU, identity, nursing research capacity, reflective case study, resilience, transnational supervision

## Abstract

**Aim:**

To critically reflect on a transnational, clinically embedded doctoral journey undertaken during and after the COVID‐19 pandemic, and to draw conceptual and systemic lessons for doctoral education and clinical academic nursing pathways.

**Background:**

Reflective accounts of doctoral study exist, yet few examine practice‐based PhDs conducted across different countries and health systems during a global crisis. This paper analyses one such pathway—enrolment at an Australian university with research embedded within the UK National Health Service—to explore resilience, identity formation, mentorship ecologies and organisational conditions that support or hinder clinical academic development.

**Method:**

Using analytic autoethnography and reflective case study logic, experiential data (field notes, supervisory records, ethics correspondence, project artefacts and publication trajectories) were synthesised with relevant scholarship. A conceptual framework, the TCAD lens, was developed to structure analysis across contexts, constraints, mechanisms and outcomes.

**Discussion:**

Four phases are outlined: starting in crisis as a senior ICU nurse, transitioning to lead educator, serving as surgical matron while implementing changes, and moving into academia to complete the thesis by publication. Dual ethics and governance procedures, contractual arrangements and GDPR‐compliant data stewardship imposed significant administrative burdens but fostered global literacy and networks. Mentorship functioned as an ecology—supportive, critical, pragmatic and strategic—evolving towards independence. COVID‐19 served as a stress test, narrowing scope while improving the feasibility and sustainability of the family member's voice reorientation intervention. Personal adversity intersected with identity development, with compassionate supervision enabling timely completion (3.7 years) and five peer‐reviewed publications.

**Conclusion:**

Transnational, clinically embedded doctoral pathways can enhance nursing research capacity but require deliberate institutional design: genuine protected time, cross‐jurisdictional support and mentorship ecosystems. The TCAD lens provides a transferable framework for educators, supervisors and health systems.

**Implications for Nursing:**

Recommendations cover programme development, cross‐border oversight, NHS–university collaborations, funding arrangements in different currencies and resilience infrastructure for clinician–researchers.

## 1 Introduction

Doctoral education in nursing now sits at the nexus of clinical service delivery, internationalisation and the altered realities of the post‐pandemic workforce. As systems continue to absorb COVID‐19's shocks, doctoral training has been both stress‐tested and reconfigured: universities rapidly shifted delivery, rebuilt academic–practice partnerships and exposed persistent gaps in equity, preparedness and technology integration, while services confronted staffing crises and burnout that re‐prioritised clinically embedded scholarship and leadership pipelines (Wu et al. 2025; Nowell et al. 2025). Together, these dynamics have reframed what a doctorate must do for nursing—advance science, translate evidence at the point of care and stabilise a strained workforce—often across borders and sectors (Sengul et al. 2025).

Reflective narratives can illuminate these lived conditions of doctoral work, but their scholarly value lies in linking personal trajectories to conceptual insight and system‐level implications. Conceptually, the literature points to three intersecting shifts: (i) a ‘post‐distance’ terrain that dissolves on‐/off‐campus binaries and normalises hybrid, translocal doctoral practice (Burford et al. 2025); (ii) a reassertion of academic–practice ecosystems (mentorship, hospital‐based research infrastructure and nurse‐scientist roles) as engines for capacity and translation (Nowell et al. 2025; Wu et al. 2025); and (iii) a workforce turn that couples doctoral development to retention, leadership and resilient care environments (Sengul et al. 2025). Systemically, these shifts imply redesigning programmes for flexible, research‐to‐practice integration; resourcing health‐system‐embedded supervision and mentorship; and aligning doctoral outputs with service metrics, equity goals and patient outcomes (Wu et al. 2025; Burford et al. 2025).

International evidence underscores this reframing. Global analyses argue that doctoral education, advanced practice and research function as an interdependent ‘three‐legged stool’ for nursing impact—yet remain unevenly developed, variably regulated and under‐resourced across WHO regions (Kim et al. 2022). Practice‐focused doctorates (e.g., DNP/DN) and innovative leadership doctorates can accelerate systems innovation, while PhDs remain central to discovery and knowledge translation; both require intentional scaffolding (funding, mentorship, role clarity) to realise career progression and system benefit (Sengul et al. 2025; Nowell et al. 2025).

COVID‐19 and its aftermath clarify the stakes. During the pandemic, postgraduate nursing students and early career clinicians faced disrupted clinical learning, rapid e‐learning pivots, inequitable access to resources and heightened psychosocial stress—conditions that both impeded progression and revealed where programmes and partnerships were fragile (Wu et al. 2025; Bange et al. 2025; Leaver et al. 2022). Post‐pandemic, burnout, turnover intention and the ‘Great Resignation’ dynamics have intensified the need for supportive work environments and nurse‐led innovation, positioning doctoral pathways as part of workforce recovery and patient safety strategies (Kurtzman et al. 2022; Burford et al. 2025).

**FIGURE 1:**
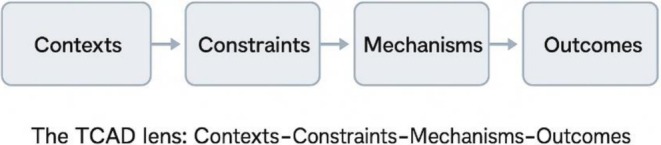
The TCAD lens.

Against this backdrop, we offer a theory‐informed reflection on a transnational, clinically embedded PhD conducted during and after the COVID‐19 period. We advance the Transnational Clinical Academic Doctorate (TCAD) as our conceptual frame (Figure 1): a doctorate deliberately designed to (a) embed the candidate within clinical service while (b) operating across national systems, standards and mentorship ecologies and (c) targeting bidirectional translation (practice‐informed research; research‐informed practice) with measurable service and patient outcomes. TCAD synthesises insights from ‘post‐distance’ doctoral formations, hospital‐based doctoral support infrastructures and global analyses of doctoral/practice/research integration, offering mechanisms for how such pathways either accelerate or stall impact (Burford et al. 2025; Feetham et al. 2025; Kim et al. 2022).

### 1.1 What This Paper Contributes

First, we theorise TCAD as a translational lens that connects an individual doctoral journey to system capabilities (capacity building, workforce stabilisation and patient‐centred improvement). Second, we distinguish pandemic effects (emergency remote pivots; clinical redeployments; disrupted competencies) from post‐pandemic imperatives (rebooting practice environments; formalising hybrid supervision and mobility; investing in mentorship and recognition structures), showing how each shaped doctoral design choices (Wu et al. 2025; Leaver et al. 2022). Third, we translate these insights into actionable implications for programme architecture (assessment of translational outputs, hybrid supervision contracts and international co‐mentorship), supervision practice (mentorship ecologies linking hospitals and universities), and NHS‐university partnership governance (shared metrics for service, equity and patient outcomes). In doing so, we argue that intentionally supported TCAD pathways can (i) accelerate research capacity via embedded pipelines and hospital‐based research ecosystems; (ii) stimulate methodological innovation by co‐producing designs responsive to complex clinical environments; and (iii) deliver patient‐centred impact through implementation‐minded scholarship aligned to service quality and equity goals (Nowell et al. 2025; Sengul et al. 2025; Feetham et al. 2025).

We pursue three aims: (1) to analyse how transnational governance, mentorship ecologies and role transitions shaped one practice‐based PhD across the pandemic divide; (2) to develop the TCAD lens and specify mechanisms through which such pathways succeed or stall; and (3) to derive design implications for doctoral programmes, supervision and NHS–university partnerships in a post‐pandemic, internationally networked nursing landscape (Kim et al. 2022).

## 2 Background and Context

### 2.1 Professional Trajectory and Topic Focus

Beginning intensive care unit (ICU) practice in 2016, I was drawn to the complexity of critical illness and, specifically, to the destabilising effects of delirium on patients and families. Despite growing awareness of delirium's burden, routine care has continued to rely heavily on pharmacological management—particularly antipsychotics—even though robust evidence shows these are largely ineffective, with current guidelines recommending against their routine use (Mart et al. 2021). Non‐pharmacological strategies, such as reorientation, sleep promotion and mobilisation, are central to best practice but are implemented unevenly across ICUs worldwide (Mart et al. 2021; Henshall et al. 2021). Within this gap, I observed the consistently calming effect of familiar family voices at the bedside. Evidence now supports this: systematic reviews show that family engagement can reduce delirium incidence by up to 24% and shorten delirium duration, even if effects on mortality and ICU length of stay remain inconclusive (Qin et al. 2022).

These experiences crystallised into a research question: can structured, standardised Family Member Voice Reorientation (FAMVR) be designed and embedded as a reproducible intervention to support delirium prevention and management in ICU?

### 2.2 Transnational Doctoral Structure and Timeline

The doctorate was undertaken at Edith Cowan University, Australia, with empirical research hosted in the UK's National Health Service (NHS). It spanned from May 2021 to December 2024, bridging the acute pandemic disruption and post‐pandemic recovery. During this period, professional roles evolved from intensive care nurse (equivalent to a senior bedside/charge nurse internationally), to critical care lead educator (comparable to a nurse educator/clinical nurse specialist), to surgical matron (akin to a nurse manager overseeing service delivery) and ultimately to an academic appointment at King's College London. This trajectory reflects the international fluidity of clinical‐academic roles and highlights the challenges of sustaining doctoral work through shifting professional contexts (Aspinall et al. 2024; Henshall et al. 2021).

### 2.3 Novelty and Contribution

This case is unusual: a transnational, clinically embedded doctorate executed during a pandemic transition, completed via multiple publications and accompanied by substantive role transitions. The contribution lies not in autobiography but in connecting lived experience to conceptual analysis of how systems enable or constrain clinical academic formation. Conceptually, integrating lived experience is increasingly recognised as essential for challenging epistemic injustice and informing innovation, but it requires translation into programmatic and systemic insight (Samra 2025; Gupta et al. 2023). By situating family integration within this translational lens, the FAMVR intervention (Johnson, Towell‐Barnard, McLean, and Ewens 2024a, 2024b) illustrates both the promise of family engagement in delirium care (Qin et al. 2022) and the persistent lack of standardisation that limits its routine adoption (Mart et al. 2021). This underscores the originality of the present study: operationalising family voices not as anecdotal practice, but as a structured, theory‐informed intervention with potential to shift methodological innovation, patient‐centred outcomes and clinical‐academic pathways in critical care nursing.

### 2.4 Methodological Positioning and Rigour

#### 2.4.1 Analytic Autoethnography and Reflective Case Study

We adopt an analytic autoethnographic approach combined with a reflective case study methodology. Here, personal experience serves as data, not as a memoir; experience is systematically generated through field notes, supervision records, ethics/governance correspondence, project artefacts and publication trail and then analysed to connect the personal, cultural and structural levels to develop concepts that extend beyond the individual (i.e., analytic generalisation/transferability) (Wall 2016; Holt 2003).

Analytic autoethnography blends evocative narrative with traditional ethnography, explicitly necessitating theory‐building, linking to literature and verification strategies while maintaining the explanatory power of the situated self (Wall 2016). First‐person writing (‘I’) is appropriate in this tradition because voice forms part of the data and analytic framework; what legitimises it is not confession but the disciplined connection of self and setting to broader systems through explicit analysis (Holt 2003; Wall 2016).

#### 2.4.2 Reflexivity, Ethics and Trustworthiness

##### 2.4.2.1 Reflexivity

We employ ongoing, in‐the‐moment reflexivity to uncover how roles and values shape knowledge production—whether as clinicians, educators, managers or academics across the UK and Australia, and to ensure these influences are open to scrutiny in the analysis (Koopman et al. 2020).

##### 2.4.2.2 Positionality

We explicitly identify an insider–outsider–in‐between stance and treat it as analytical material, focusing on power, representation and the ‘outsider‐within’ issue typical of practice‐embedded research; this enhances more credible interpretations of clinical–academic cultures (Kamlongera 2021).

##### 2.4.2.3 Ethics of Representation

Beyond formal approvals, we mitigate the ‘representation crisis’ by anonymising institutions and individuals where appropriate, negotiating disclosures and linking experience to theory to reduce the risks of therapeutic self‐writing and inadvertent harm (Holt 2003; Wall 2016).

##### 2.4.2.4 Trustworthiness

We utilise triangulation across diverse data sources; maintain an audit trail (documented decisions, dated memos, artefacts); and engage in peer debriefing and mentorship dialogues to challenge initial sense‐making (Holt 2003; Wall 2016). By combining disciplined reflexivity (visual and narrative prompts, memoing), multiple data types and an auditable chain of evidence, we address autoethnography's verification concerns while strengthening theory‐linked claims with transferability rather than sample‐based generalisability (Koopman et al. 2020; Holt 2003; Wall 2016).

#### 2.4.3 Conceptual Scaffolding

We organise the analysis around three strands that prepare for the TCAD lens:

##### 2.4.3.1 Resilience as a Dynamic Process

Rather than a trait, resilience is regarded as an ongoing process—continuous negotiation of identities, roles and power across shifting contexts (pandemic/post‐pandemic; service/academy). Autoethnography demonstrates this dynamism by illustrating how situated choices and constraints are made visible and theorised, not merely narrated (Wall 2016; Kamlongera 2021).

##### 2.4.3.2 Professional Identity Formation

The transition from clinician to clinical academic is examined as identity work at the self–culture interface. Analytic autoethnography involves connecting these changes to organisational norms and discourses, while avoiding ‘self‐absorption’ through explicit theorising and verification strategies (Holt 2003; Wall 2016).

##### 2.4.3.3 Organisational and International Higher Education Frameworks

Positionality and reflexive methods are employed to examine transnational supervision, governance, mentorship ecologies and capacity‐building logics—demonstrating how experiences within one system are analytically linked to mechanisms that are likely to recur elsewhere (Koopman et al. 2020; Kamlongera 2021).

### 2.5 Methodological Implication

Framing the study as an analytic autoethnography combined with reflection allows us to (a) treat experience as data, (b) enhance findings through concept development and transferability and (c) address known critiques (legitimisation, ‘narcissism’) through rigour strategies and ethical representation. This approach has already been successfully employed in nursing, health and social research contexts (Koopman et al. 2020; Holt 2003; Wall 2016; Kamlongera 2021).

## 3 Data Sources

### 3.1 The TCAD Lens

#### 3.1.1 Why TCAD Is Needed

Doctoral education in nursing is increasingly expected to generate both knowledge production and knowledge translation, yet existing models often isolate research training from clinical service or international engagement (Kim et al. 2022; Aspinall et al. 2024). The pandemic further highlighted the fragility of doctoral support structures and underscored the urgent need to integrate training with workforce resilience and patient outcomes (Wu et al. 2025; Kurtzman et al. 2022). While frameworks for clinical academic careers exist, they remain unevenly applied and often limited to a national level, restricting their ability to respond to the global realities of mobility, governance and cross‐system partnerships (Henshall et al. 2021; Feetham et al. 2025). These gaps justify a new integrative perspective.

#### 3.1.2 What TCAD Is

The TCAD is proposed as a conceptual framework to encompass doctoral pathways that are concurrently (Figure 1):

1. Transnational—spanning systems, governance structures and supervisory ecologies across borders, embedding comparative and mobile dimensions into doctoral training (Kim et al. 2022).
2. Clinically embedded—integrated within healthcare organisations and professional roles, combining doctoral inquiry with frontline service delivery and patient care (Feetham et al. 2025; Nowell et al. 2025).
3. Translational—designed to promote bidirectional knowledge exchange: practice‐informed research questions and research‐informed practice interventions, with patient and workforce outcomes as endpoints (Sengul et al. 2025; Gupta et al. 2023).

Unlike descriptive accounts of ‘doctoral experiences’, TCAD positions the doctoral pathway itself as a driver of system change, embedding scholarship in ways that intentionally focus on service resilience, innovation and equity.

#### 3.1.3 Conceptual Scaffolding

Three strands underpin TCAD:

- Resilience as a process: resilience is not a fixed trait but a dynamic negotiation across individual, organisational and systemic levels; TCAD operationalises this by tracking how doctoral candidates adapt and reconfigure roles under structural constraints (Wall 2016; Kamlongera 2021).
- Professional identity formation: TCAD highlights the transition from clinician to clinical academic as a process of identity development influenced by supervision, mentorship and governance, rather than simply a linear career progression (Holt 2003; Samra 2025).
- Organisational and transnational higher education frameworks: TCAD emphasises how cross‐system supervision, governance and partnership arrangements either facilitate or hinder doctoral progression and research capacity‐building (Koopman et al. 2020; Aspinall et al. 2024).

#### 3.1.4 Contribution and Practical Implications

The TCAD lens offers a transferable framework for doctoral education with direct implications:

- Programme design: Incorporate hybrid, cross‐system structures (international co‐supervision, embedded clinical placements, transferable credit/recognition) to ensure doctoral pathways combine research training with service delivery and mobility (Kim et al. 2022; Aspinall et al. 2024).
- Supervision and mentorship: Develop networks of supervision that connect academic and clinical mentors, creating triangulated support to reduce attrition and strengthen identity development as clinical academics (Koopman et al. 2020; Henshall et al. 2021).
- NHS–university partnerships: Develop shared governance and accountability frameworks, adopt common metrics for research capacity, workforce retention and patient outcomes—so that doctoral outputs are recognised both academically and clinically (Feetham et al. 2025; Nowell et al. 2025).
- Equity and sustainability: utilise transnational arrangements to address inequities in doctoral access and resourcing, while aligning projects with resilience‐building at individual, organisational and system levels (Kurtzman et al. 2022; Wall 2016).

In summary, the TCAD lens integrates transnational, clinically embedded and translational aspects of doctoral education into a framework that highlights resilience, identity development and governance. Having outlined its conceptual boundaries, the next step is to apply TCAD as an analytical framework to a reflexive case study. In doing so, personal experiences are regarded as data, but the analysis goes beyond recounting narratives to explore how governance, mentorship, role transitions and pandemic/post‐pandemic circumstances influenced doctoral formation. This dual approach, reflection grounded in theory, ensures that what follows is not autobiography but a reflexive analysis offering transferable insights for programme design, supervision and system‐level collaborations.

### 3.2 Applying the TCAD Lens: Reflexive Analysis

The reflexive analysis is based on the TCAD framework, which offers a conceptual structure for connecting lived doctoral experiences to broader systemic frameworks. This aligns with analytic autoethnography, where personal experience is regarded as data but examined for conceptual development and transferability rather than solely self‐report (Holt 2003; Wall 2016). The TCAD framework facilitated reflection on four major career transitions: senior ICU nurse, lead educator, surgical matron and academic secondment, while situating these within doctoral training, international supervision and post‐pandemic service contexts.

#### 3.2.1 Commencing in Crisis (2021): Senior ICU Nurse

Working in an expanded ICU for acute respiratory distress syndrome (ARDS) during the COVID‐19 pandemic, I managed 12‐h shifts while also dedicating days off to research, drafting the literature review and proposal. As ICU nurses worldwide reported, the pandemic increased workload, moral distress and psychosocial pressure (Nikbakht Nasrabadi et al. 2022). Mentoring redeployed staff not only showed leadership under pressure but also facilitated later role transitions, reflecting how PhD‐trained nurses develop leadership skills even in challenging clinical environments (van Dongen and Hafsteinsdóttir 2022). Research activities were pushed to the margins, revealing the structural tensions between clinical duties and academic progress.

#### 3.2.2 Becoming a Lead Educator: Capability Building Under Pressure

The promotion to lead educator involved responsibilities for onboarding both new and overseas nurses without formal critical care certification. As highlighted in scoping reviews, transitions from clinician to educator roles are often destabilising and lack sufficient support, with limited preparatory structures (Halton et al. 2024). Protected research time proved illusory as managerial demands increased, and doctoral engagements in Perth, Australia, occurred at inconvenient hours due to time‐zone differences. This reflects the experiences of distance doctoral students, whose sense of institutional belonging is fragile, and support systems often lag behind diverse modes of enrollment (McChesney et al. 2025). The ability to maintain research momentum despite fatigue demonstrates resilience as a dynamic process, negotiated between individual agency and structural pressures.

#### 3.2.3 Surgical Matron: Ethics, Proximity and Isolation

Transitioning to a matron role overseeing surgical wards reduced conflicts of interest in implementation but increased operational workload. Maintaining a presence in ICU for FAMVR uptake reflected the blurred clinician researcher roles discussed in autoethnographic nursing scholarship (Koopman et al. 2020). However, isolation deepened: few nurse leaders within the Trust pursued doctorates, echoing findings that clinical academic nurses often feel unsupported within hospital frameworks (van Dongen and Hafsteinsdóttir 2022). The pivotal supervisory question—Professor or Director of Nursing?—crystallised tensions in professional identity development, a common turning point in doctoral journeys where leadership paths diverge (Halton et al. 2024).

#### 3.2.4 Entering Academia: Focus, Accountability and Completion

Secondment to King's College London immersed me in an environment where doctoral progress was expected and normalised. While the academic workload was substantial—covering module leadership and postgraduate teaching—alignment with scholarly goals supported timely completion. These experiences mirror broader trends seen in nurses shifting from clinical to academic roles, where identity renegotiation, structural support and scholarly integration are vital for success (Greenway et al. 2025). By the end, four peer‐reviewed papers had been published and a fifth was accepted, demonstrating how transnational doctorates can encourage methodological innovation, leadership growth and patient‐centred impact when sufficiently supported.

## 4 Overview of the Issues

### 4.1 Governance and Ethical Complexities in Transnational Research

Carrying out doctoral research across Australia and the UK required fluency in two distinct yet overlapping systems of research governance. In Australia, Human Research Ethics Committees (HRECs) have evolved from peer‐review bodies into ‘devolved regulators’, responsible for product safety, waivers of consent and the inclusion of incapacitated participants under state guardianship law (Eckstein et al. 2025). In contrast, in the UK, oversight is managed by the Health Research Authority (HRA) and its Research Ethics Committees, with a continual focus on efficiency and proportionality in review processes. However, inefficiencies, duplication and disproportionate review of low‐risk studies have been shown to delay or even hinder valuable research (Glasziou et al. 2021).

This transnational doctorate, therefore, encountered three levels of complexity (Table 1):

1. Reciprocal ethics approvals (HRA in the UK and HREC at Edith Cowan University in Australia).
2. Contractual governance between the host university and the NHS Trust to regulate intellectual property, data custody and indemnity.
3. Data governance compliance, especially strict adherence to GDPR, which mandated NHS‐resident storage of patient data and clear separation from university systems (Lyle et al. 2023; Xia et al. 2024).

**TABLE 1:** Transnational challenges and benefits.

Domain	Challenge (UK–AU)	Mitigation/Mechanism	Benefit/Outcome
Ethics & governance	Dual approvals (HRA, HREC)	Reciprocal pathway; early parallel prep	Faster subsequent approvals; literacy in both systems
Contracts & funding	Cross‐currency, cross‐institution transfers	Early legal liaison; buffer for FX	Predictable resourcing; admin competence
Data & GDPR	Data residency on NHS servers	Segregated storage; SOPs	Compliance; trust with the site
Time zones	Perth–London coordination	Fixed windows; asynchronous updates	Global collaboration skills

The challenges were heightened by the research focus on critically ill ICU patients, who constitute a vulnerable group with fluctuating decisional capacity. Both the UK and Australia incorporate specific safeguards for such participants: in the UK through HRA and Human Tissue Act requirements related to ‘qualifying consent’, and in Australia via HREC and state‐based guardianship approvals (Eckstein et al. 2025; Lyle et al. 2023). While these safeguards are essential, they have led to prolonged timelines and administrative processes, reflecting broader critiques that governance structures sometimes prioritise compliance over context‐sensitive ethical practice (Lyle et al. 2023).

Yet, negotiating these requirements also yielded significant benefits. The necessity to satisfy two national systems fostered a depth of regulatory fluency uncommon in single‐site doctorates. It broadened mentorship networks across the UK, Australia and later the US, creating a platform for methodological transferability. As others have argued, proportionate and streamlined governance systems are essential to reduce waste in research and accelerate patient benefits (Glasziou et al. 2021). In this case, the deliberate design of protocols that met the highest standards across both jurisdictions meant the study was inherently adaptable and transferable across centres, enhancing its potential contribution to international nursing research capacity.

### 4.2 Mentorship as Ecology: Complementarity and Independence

Supervision during the doctorate operated not as a single dyadic relationship but as an ecology of differentiated roles, each contributing complementary expertise. A principal supervisor offered person‐centred, holistic support; an associate supervisor provided critical challenge; a UK‐based supervisor ensured NHS alignment and pragmatic translational focus; and an external senior mentor offered strategic methodological and scholarly guidance. Such arrangements align with the idea of mentorship ecosystems, where effective development relies on a constellation of mentors embedded in interdependent systems rather than on a single relationship (Hund et al. 2018; Mondisa et al. 2021).

This ecology also reflected evolving reciprocity. Early in candidature, dependence on detailed feedback predominated; over time, autonomy increased, culminating in leading journal revisions end‐to‐end with supervisors verifying near‐final drafts. Such a transition aligns with developmental models of mentoring that emphasise the gradual shift from dependence to independence, and with reverse mentoring principles, where doctoral candidates may also contribute new expertise (e.g., digital scholarship, open science) to senior academics (Pizzolato and Dierickx 2022).

Tensions—such as fluctuating feedback pace in the final year—were genuine, but these coincided with the development of scholarly independence, demonstrating how friction within mentoring relationships can be productive. As STEM‐ME research indicates, thriving ecosystems are not free from strain but manage support, critique and independence through stewardship at various levels of the system (Mondisa et al. 2021).

For the TCAD lens, this supervisory ecology was essential. Transnational, clinically embedded doctorates require multi‐layered mentorship: ensuring clinical relevance, cross‐system governance fluency, methodological innovation and international scholarly alignment. Conceptualising supervision as an ecology shows how doctoral paths are supported by complementary roles and evolving reciprocity, ultimately scaffolding the shift from clinician to independent clinical academic.

### 4.3 COVID‐19 as a Stress Test: Scope, Feasibility and Sustainability

An initially ambitious goal, to develop, implement and prepare the FAMVR intervention for RCT, was necessarily narrowed down to co‐design and a pilot within a single Trust. Pandemic‐related issues such as staff redeployment, limited availability and institutional restrictions significantly influenced recruitment and assessment. Such disruption to feasibility mirrors broader reports that ICU clinical trials experienced start–stop cycles, lower recruitment and reallocation of research staff during COVID‐19, even when protocols remained open (Chlan et al. 2023).

Embedding the intervention required visible presence, education and trust‐building with nurses wary of additional workload. This dynamic reflects the ICU strain literature, where bed expansion, non‐ICU staff redeployment and heightened nurse‐to‐patient ratios were directly associated with complications and adverse outcomes (Kohler et al. 2025). Paradoxically, these constraints sharpened the translational edge of the doctorate: implementation was tested under conditions of maximum strain, forcing the intervention to be resource‐sensitive, adaptable and sustainable under pressure.

At the same time, the pandemic revealed dual legacies. On one hand, constraints limited enrolment and slowed trial readiness; on the other, they catalysed methodological innovation and more realistic assumptions for multicentre scale‐up. This paradox is noted in broader scholarship: while the pandemic disrupted non‐COVID research pathways, it also generated rapid innovations in trial design, governance flexibility and cross‐centre collaboration (Hermann et al. 2023).

From a TCAD perspective, the stress test demonstrated the importance of aligning clinical services with doctoral work. My dual role as ICU clinician and doctoral researcher highlighted both the weaknesses of strained health systems and the avenues through which doctoral projects can adapt to these challenges. COVID‐19 thus served as a crucible: limited in scope but enriching in sustainability, embedding feasibility into the intervention and emphasising the translational value of clinically embedded doctoral research (Tu et al. 2023; Börgeson et al. 2021).

## 5 Findings

### 5.1 Personal Adversity, Resilience and Identity Formation

Personal upheaval—living apart from family and the breakdown of a marriage during the final year—intersected with academic demands. Choosing perseverance over pause, I reframed adversity as fuel for completing my doctorate in 3 years and 7 months. Such reframing aligns with findings that resilience in doctoral students is not static but a dynamic process, built through the ongoing negotiation of adversity, agency and support networks (Wu 2022).

Compassionate supervision validated vulnerability and fostered boundary‐setting, emphasising how supportive supervisory environments can mitigate risks of poor wellbeing and mental health issues that are still widespread among PhD students globally (Levecque et al. 2017). Resilience here was not solely about personal perseverance but also involved the dynamic interaction of institutional support and chosen family networks, reflecting evidence that identity formation during doctoral study is co‐constructed through academic, personal and relational aspects (Zhao et al. 2021).

At the same time, the upheaval deepened my understanding of ‘family’ in both research and life, emphasising the importance of family‐centred approaches to ICU brain dysfunction while reaffirming the vital role of chosen family and academic community. Literature on PhD identity and well‐being shows that adversity can stimulate positive growth, with doctoral students reporting increased clarity of purpose and resilience when adversity is seen as part of their identity development (Martínez‐García et al. 2024; Sverdlik et al. 2018).

**FIGURE 2:**
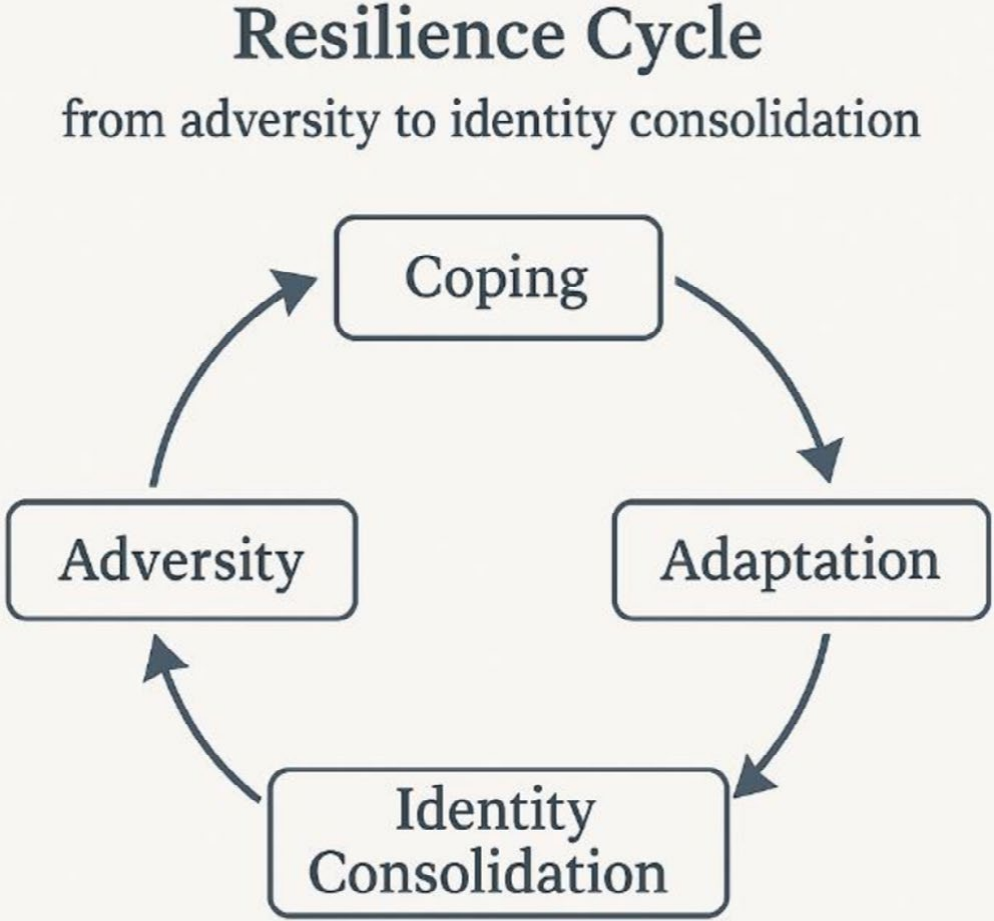
Resilience cycle from adversity to identity consolidation.

My trajectory exemplifies how the TCAD lens embeds doctoral identity formation within translational, clinically integrated contexts: adversity was not incidental but essential for resilience‐building, identity development and translational insight (Figure 2). Such reflections highlight the potential for doctoral journeys to produce transferable resilience frameworks, which benefit both the individual researcher and the wider system of clinical academic training (Stubb et al. 2011).

### 5.2 Outputs, Recognition and Reciprocal Mentoring

The thesis by publication resulted in five peer‐reviewed papers: a scoping review of non‐pharmacological delirium interventions, a qualitative co‐design study of FAMVR, a mixed‐methods pilot implementation and two methodological contributions (application of the Double Diamond design and interpretive descriptive insights from implementation) (Table 2). Publishing four papers during candidature, prior to submission, provided external validation and momentum. This aligns with work on doctoral recognition, which emphasises that publication is both a symbolic marker of scholarly worth and a practical part of building academic identity (Cuthbert and Spark 2008).

**TABLE 2:** Thesis publications and contributions.

#	Article type	Focus	Distinctive contribution	Publication status
1	Scoping review	Non‐pharmacological ICU delirium interventions (Johnson, Towell‐Barnard, McLean, and Ewens 2024)	Synthesises gaps; frames FAMVR rationale	Published
2	Methodology	Double Diamond co‐design (Johnson, Towell‐Barnard, McLean, Robert, and Ewens 2024)	First application in ICU intervention development	Published
3	Qualitative	Co‐design of FAMVR (Johnson, Towell‐Barnard, McLean, and Ewens 2024a)	Lived‐experience integration; acceptability	Published
4	Mixed methods	Pilot implementation of FAMVR (Johnson, Towell‐Barnard, McLean, and Ewens 2024b)	Feasibility, fidelity, preliminary outcomes	Published
5	Methodological insights	Interpretive descriptive evaluation (Johnson et al. 2025)	Practical guidance for implementation studies	Published

Recognition on the Executive Dean's List signifies academic excellence. However, such markers of success are part of broader ‘rules of recognition’ that early career academics must navigate—balancing competitive achievement talk with authenticity, loyalty and credibility within evaluative cultures (Nästesjö 2023). In this context, ECU's flexible thesis‐by‐publication policy allowed coherence to take priority over strict acceptance timing, reducing pressure while maintaining ambition. Flexibility in institutional recognition structures has been shown to improve doctoral satisfaction and enthusiasm for academic careers, especially when combined with strong supervision and peer support (Yang and Cai 2022).

Navigating journal peer review—from initial rejections to eventual acceptance in high‐tier outlets—fostered resilience and rhetorical clarity. Literature highlights that such iterative processes are not just technical but also identity‐shaping, as doctoral students struggle to ‘feel like academic writers’ while learning to adopt the norms of their disciplines (Cotterall 2011). Within the TCAD framework, publication thus served not merely as output but as translational training, immersing me in disciplinary debates while enhancing my ability to communicate ICU practice insights to scholarly and clinical audiences.

Mentorship during this phase was reciprocal. Alongside receiving guidance, I supported at least 24 nurses in advancing towards leadership, master's and doctoral pathways. This illustrates how mentoring can flow both ways: doctoral candidates can act as both mentees and mentors, strengthening professional identity and community capacity (Nästesjö 2023). Such reciprocity embodies the TCAD principle of linking individual doctoral progress with collective clinical academic development, sustaining both systems.

### 5.3 From Experience to System Design: Thematic Insights Through the TCAD Lens

Viewed through the TCAD perspective, this reflective account reveals several cross‐cutting lessons for designing clinical academic pathways. First, the *myth of protected time* repeatedly surfaces: promotions within clinical services often decrease rather than increase research capacity unless time is both contractually protected and operationally respected. This aligns with broader evidence that doctoral productivity depends not only on individual effort but also on the structural protection of research time within health systems (Levecque et al. 2017; Aspinall et al. 2024). Second, the *invisibility of clinical academic labour* remains a persistent challenge. Transnational governance and dual‐system compliance with NHS, GDPR and HREC requirements involve significant unseen workload that is rarely reflected in milestones or workload models (Eckstein et al. 2025; Glasziou et al. 2021). This underscores the need for explicit recognition of governance fluency as both intellectual labour and system‐level competence.

Third, the analysis highlights *institutional literacy as a fundamental competence*. Early mastery of ethics, contracts and policy frameworks reduced waste and protected integrity, aligning with calls for doctoral programmes to incorporate policy and governance training alongside methodological skills (Lyle et al. 2023). Fourth, *mentorship as an ecosystem* proved essential. Progress relied on a deliberately assembled supervisory team that provided supportive, critical, pragmatic and strategic input. This aligns with mentoring ecosystem models, which argue that doctoral development thrives within constellations rather than dyads (Hund et al. 2018; Mondisa et al. 2021).

Fifth, *visibility and networking* remained crucial for maintaining momentum. A professional presence, especially on platforms like LinkedIn, not only built social capital but also normalised doctoral work within clinical teams and attracted collaborators, illustrating how doctoral identity formation increasingly intersects with public scholarship and professional networking (Cotterall 2011; Nästesjö 2023). Sixth, resilience was shown to be both *relational and structural*. Personal perseverance was important, but completion depended on compassionate supervision, peer support and employer collaboration—findings aligned with evidence that resilience in PhD students is a dynamic, socially rooted process rather than merely an individual trait (Wu 2022; Martínez‐García et al. 2024).

Finally, the TCAD lens highlights the importance of *rethinking impact*. Transitioning from bedside practice to education and scholarship broadens influence through students and systems, rather than solely through individual patients. This aligns with international calls to expand nursing impact via research, leadership and pedagogy (Kim et al. 2022; Sengul et al. 2025). Additionally, *transnational literacy* has become a key asset: graduates who are fluent across different jurisdictions are uniquely able to accelerate knowledge transfer and application across health systems, promoting equity and resilience in global nursing scholarship (Nowell et al. 2025; Kurtzman et al. 2022).

## 6 Discussion

This paper expands scholarship on doctoral resilience, clinical academic pathways and transnational supervision by providing a conceptually grounded, practice‐oriented analysis of a cross‐border PhD completed during the COVID‐19 transition. The TCAD perspective demonstrates how contexts and constraints interact with mechanisms (mentorship ecologies, visibility, boundary setting, institutional literacy) to produce outcomes relevant to capacity building. Importantly, it shows how pandemic‐driven scope adjustments—often seen as obstacles—can paradoxically improve fidelity and sustainability by fostering resource‐sensitive, adaptable design.

Across the literature, the gap between nurses' clinical academic pathways and those of doctors and dentists remains pronounced. For medicine and dentistry, clinical academia is well embedded, with established pathways, formal structures and consistent funding streams (Funston et al. 2015; Lopes et al. 2017). In contrast, for nurses and allied health professionals, the pathway remains fragmented, underfunded and culturally undervalued (Trusson et al. 2019; Bluemel et al. 2024). This disparity reflects systemic inequalities: where doctors' research is integrated into training from the start, nurses and midwives often face resistance from managers prioritising service delivery, lack protected time and have limited role models. The result is what Trusson et al. (2019) describe as a ‘pyramid’ structure, with many able to begin but few able to progress beyond the doctorate into stable clinical academic positions.

Based on this case and broader evidence, we assert that clinical academic doctorates for nurses require targeted support: enforceable protected time embedded in contracts, administrative infrastructure for cross‐jurisdictional ethics and data governance, dedicated funding streams that reflect international costs and contracts and supervisor development for cross‐border mentoring. The TCAD perspective introduces novelty by viewing these not as temporary fixes but as structural conditions for translation: enabling doctoral outputs to expand into clinical and educational systems. Literature on barriers and facilitators supports these claims, highlighting the need for institutional recognition of educational and clinical research as legitimate, and the development of networks and mentorship ecologies to sustain careers (Bluemel et al. 2024).

This study also highlights the broader value of clinical academics. Beyond individual resilience, the doctorate develops transferable skills: mentoring future researchers, integrating innovation into ICU practice and informing doctoral design for policy and institutions. These align with findings that nurse clinical academics provide patient benefits, organisational efficiencies and role‐model visibility that go beyond individual projects (Trusson et al. 2019). However, without structural support, such contributions risk being lost as nurses are pushed from dual roles into either service‐only or academic‐only tracks, weakening the translational bridge that TCAD aims to sustain.

Limitations include the single‐case design and reflexive bias, although credibility is supported by triangulation of artefacts and alignment with established scholarship. Transferability is strengthened by situating the analysis within broader studies across nursing, medicine and allied health. Future research should examine the TCAD lens across disciplines and assess institutional interventions—such as transnational doctoral coordinators or bespoke funding mechanisms—for their effects on completion and publication outcomes.

### 6.1 Implications for Global Nursing Practice, Education, Research and Policy

This analysis highlights that supporting clinical academic doctorates in nursing requires coordinated efforts across practice, education, research and policy. For *practice*, employers must formalise protected time in contracts, create dual clinical–academic roles with backfill, and explicitly acknowledge transnational administration as legitimate workload; such measures sustain research productivity and prevent attrition (Trusson et al. 2019). For *education*, universities should provide cross‐border ethics and contracting toolkits, expand thesis‐by‐publication models with structured writing support, and train supervisors in transnational mentorship, recognising that HDR peer communities across time zones enhance belonging and reduce isolation (McChesney et al. 2025). For *research*, clinical academic programmes should adopt mentorship ecology planning, support early publications and value administrative competence alongside methodological expertise, embedding implementation science from the outset to reinforce translational impact (Hund et al. 2018; Mondisa et al. 2021). For *policy*, funders and regulators need to harmonise reciprocal ethics pathways, streamline GDPR‐compliant data sharing agreements, and develop mechanisms to buffer currency exchange, while incentivising NHS–university partnerships with measurable outcomes (Eckstein et al. 2025; Glasziou et al. 2021). Collectively, these actions turn the TCAD perspective into tangible reforms that can bolster doctoral resilience, reduce inequities with medicine and dentistry (Funston et al. 2015), and accelerate the integration of nursing scholarship into clinical systems worldwide.

## 7 Conclusion

This case shows that transnational, clinically integrated doctoral pathways can succeed even during systemic disruption when mentorship networks, institutional understanding and protected time are carefully coordinated. The TCAD perspective offers a practical framework for educators, supervisors and health system leaders to create support systems that turn challenges into opportunities and opportunities into meaningful outcomes. In this example, such structuring allowed for timely completion, the development of a coherent set of peer‐reviewed publications and the strengthening of a lasting scholarly identity focused on family‐oriented ICU survivorship.

## Funding

This doctoral project was supported by the Australian Government Research Training Programme, which provided tuition coverage, a stipend and research cost support.

## Disclosure

Author positionality and reflexivity statement: I write as a Black, internationally trained ICU clinician–educator who completed a transnational PhD while holding NHS leadership roles and subsequently securing an academic appointment in the UK. My commitments to family‐centred care, equity in clinical education and compassionate supervision shape my framing and analysis. Reflexive journaling and regular consultation with mentors are employed to identify blind spots and to resist over‐attributing outcomes to personal perseverance in contexts where structural supports—or their absence—were decisive. This reflexive stance aligns with analytic autoethnography, where personal experience is regarded as data used to shed light on wider cultural and systemic dynamics.

## Ethics Statement

The doctoral project received ethics approval from the UK Health Research Authority and the Edith Cowan University Human Research Ethics Committee through reciprocal arrangements and fully adhered to GDPR requirements. All patient data were stored on a secure NHS infrastructure and managed according to NHS governance protocols. This discursive reflection only draws on non‐identifiable project artefacts (protocols, supervision records, correspondence and publications) and personal field notes, ensuring compliance with both ethical and data governance standards.

## Conflicts of Interest

The author declares no conflicts of interest.

## Data Availability

Data sharing does not apply to this article, as no new datasets were created or analysed during the current study.

## References

[jan70421-bib-0001] Aspinall, C. , J. Slark , J. Parr , B. J. Pene , and M. Gott . 2024. “The Role of Healthcare Leaders in Implementing Equitable Clinical Academic Pathways for Nurses: An Integrative Review.” Journal of Advanced Nursing 80, no. 8: 3119–3133. 10.1111/jan.16043. 38186212

[jan70421-bib-0002] Bange, J. , W. Gao , and K. Crawford . 2025. “Graduate Nurses' Experience of Support, Training, and Education During the COVID‐19 Pandemic: A Qualitative Study.” Collegian 32, no. 2: 120–127. 10.1016/j.colegn.2025.02.002.

[jan70421-bib-0003] Bluemel, A. H. , O. E. Burton , B. Burford , et al. 2024. “Barriers and Facilitators to Establishing a Clinical Academic Career in Clinical Education Research in the UK: A Focus Group Study.” Medical Teacher 46, no. 10: 1369–1377. 10.1080/0142159X.2024.2308783. 38359431

[jan70421-bib-0004] Börgeson, E. , M. Sotak , J. Kraft , G. Bagunu , C. Biörserud , and S. Lange . 2021. “Challenges in PhD Education due to COVID‐19: Disrupted Supervision or Business as Usual? A Cross‐Sectional Survey of Swedish Biomedical Sciences Graduate Students.” BMC Medical Education 21: 294. 10.1186/s12909-021-02727-3. 34022871 PMC8140581

[jan70421-bib-0005] Burford, J. , K. McChesney , L. Frick , and T. Khoo . 2025. “Conceptualising Distance Doctoral Study After COVID‐19: Are We Post‐Distance Now?” Distance Education 46, no. 3: 496–513. 10.1080/01587919.2024.2388216.

[jan70421-bib-0006] Chlan, L. L. , M. F. Tracy , J. Ask , A. Lal , and J. Mandrekar . 2023. “The Impact of the COVID‐19 Pandemic on ICU Clinical Trials: A Description of One Research Team's Experience.” Trials 24: 321. 10.1186/s13063-023-07355-4. 37165383 PMC10172070

[jan70421-bib-0007] Cotterall, S. 2011. “Doctoral Students Writing: Where's the Pedagogy?” Teaching in Higher Education 16, no. 4: 413–425. 10.1080/13562517.2011.560381.

[jan70421-bib-0055] Cuthbert, D. , and C. Spark . 2008. “Getting a GRiP: Examining the Outcomes of a Pilot Program to Support Graduate Research Students in Writing for Publication.” Studies in Higher Education 33, no. 1: 77–88. 10.1080/03075070701794841.

[jan70421-bib-0008] Eckstein, L. , J. C. Kaldor , and C. Stewart . 2025. “The Role of HRECs in Regulating Medical Research: From Peer Review to Regulation.” Monash Bioethics Review 43, no. 2: 204–224. 10.1007/s40592-025-00248-z. 40372559 PMC12202549

[jan70421-bib-0009] Feetham, S. , K. P. Kelly , C. D. Colson , et al. 2025. “A Hospital Resource for Nurses Navigating Doctoral Education and Beyond.” Journal of Nursing Administration 55, no. 2: 89–96. 10.1097/NNA.0000000000001535. 39807900 PMC11913225

[jan70421-bib-0010] Funston, G. , C. Cerra , and D. Kirkham . 2015. “The Road to a Clinical Academic Career.” British Medical Journal 350: h3445. 10.1136/bmj.h786.26116693

[jan70421-bib-0011] Glasziou, P. , A. M. Scott , I. Chalmers , S. E. Kolstoe , and H. T. Davies . 2021. “Improving Research Ethics Review and Governance Can Improve Human Health.” Journal of the Royal Society of Medicine 114, no. 12: 556–562. 10.1177/01410768211051711. 34761994 PMC8722776

[jan70421-bib-0012] Greenway, M. , E. Belita , P. Baxter , J. Pierazzo , and S. A. Boamah . 2025. “Exploring Nurses' Experiences Transitioning From Clinicians to Professors at Ontario Colleges.” Canadian Journal of Nursing Research 57, no. 2: 256–266. 10.1177/08445621251320708. PMC12086282 40239171

[jan70421-bib-0013] Gupta, V. , C. Eames , L. Golding , et al. 2023. “Understanding the Identity of Lived Experience Researchers and Providers: A Conceptual Framework and Systematic Narrative Review.” Research Involvement and Engagement 9, no. 26: 1–21. 10.1186/s40900-023-00439-0. 37095587 PMC10127294

[jan70421-bib-0014] Halton, J. , C. Ireland , and B. Vaughan . 2024. “The Transition of Clinical Nurses to Nurse Educator Roles—A Scoping Review.” Nurse Education in Practice 78: 104022. 10.1016/j.nepr.2024.104022. 38875844

[jan70421-bib-0015] Henshall, C. , O. Kozlowska , H. Walthall , A. Heinen , R. Smith , and P. Carding . 2021. “Interventions and Strategies Aimed at Clinical Academic Pathway Development for Nurses in the United Kingdom: A Systematised Review of the Literature.” Journal of Clinical Nursing 30, no. 11–12: 1502–1518. 10.1111/jocn.15657. 33434295

[jan70421-bib-0016] Hermann, B. , S. Benghanem , Y. Jouan , A. Lafarge , A. Beurton , and the ICU French FOXES Study Group . 2023. “The Positive Impact of COVID‐19 on Critical Care: From Unprecedented Challenges to Transformative Changes.” Annals of Intensive Care 13: 28. 10.1186/s13613-023-01118-9. 37039936 PMC10088619

[jan70421-bib-0017] Holt, N. L. 2003. “Representation, Legitimation, and Autoethnography: An Autoethnographic Writing Story.” International Journal of Qualitative Methods 2, no. 1: 18–28. 10.1177/160940690300200102.

[jan70421-bib-0018] Hund, A. K. , A. C. Churchill , A. M. Faist , et al. 2018. “Transforming Mentorship in STEM by Training Scientists to Be Better Leaders.” Ecology and Evolution 8, no. 19: 9962–9974. 10.1002/ece3.4527. 30397439 PMC6206201

[jan70421-bib-0019] Johnson, G. U. , A. Towell‐Barnard , C. McLean , and B. Ewens . 2024. “Delirium Prevention and Management in an Adult Intensive Care Unit Through Evidence‐Based Nonpharmacological Interventions: A Scoping Review.” Collegian 31, no. 4: 232–251. 10.1016/j.colegn.2024.05.001.

[jan70421-bib-0020] Johnson, G. U. , A. Towell‐Barnard , C. McLean , and B. Ewens . 2024a. “The Development of a Family‐Led Novel Intervention for Delirium Prevention and Management in the Adult Intensive Care Unit: A Co‐Design Qualitative Study.” Australian Critical Care 38: 101088. 10.1016/j.aucc.2024.07.076. 39129064

[jan70421-bib-0021] Johnson, G. U. , A. Towell‐Barnard , C. McLean , and B. Ewens . 2024b. “The Implementation and Evaluation of a Family‐Led Novel Intervention for Delirium Prevention and Management in Adult Critically Ill Patients: A Mixed‐Methods Pilot Study.” Nursing in Critical Care 29, no. 6: 1–11. 10.1111/nicc.13210. PMC12224221 39617957

[jan70421-bib-0022] Johnson, G. U. , A. Towell‐Barnard , C. McLean , G. Robert , and B. Ewens . 2024. “Co‐Designing a Digital Family‐Led Intervention for Delirium Prevention and Management in Adult Critically Ill Patients: An Application of the Double Diamond Design Process.” International Journal of Nursing Studies 160: 104888. 10.1016/j.ijnurstu.2024.104888. 39303642

[jan70421-bib-0023] Johnson, G. U. , A. Towell‐Barnard , C. McLean , G. Robert , and B. Ewens . 2025. “Methodological Insights From Evaluating a Family Member's Voice Reorientation Programme: An Interpretive Descriptive Approach.” Nursing Open 12, no. 9: e70291. 10.1002/nop2.2567. 40887932 PMC12399836

[jan70421-bib-0024] Kamlongera, M. I. 2021. “‘So What's Arts Got to Do With It?’ An Autoethnography of Navigating Researcher Positionality While Co‐Creating Knowledge.” Qualitative Research 23, no. 3: 651–667. 10.1177/14687941211045611.

[jan70421-bib-0025] Kim, M. J. , H. McKenna , P. Davidson , et al. 2022. “Doctoral Education, Advanced Practice and Research: An Analysis by Nurse Leaders From Countries Within the Six WHO Regions.” International Journal of Nursing Studies Advances 4: 100094. 10.1016/j.ijnsa.2022.100094. 38745635 PMC11080457

[jan70421-bib-0026] Kohler, K. , T. De Corte , M. Greco , et al. 2025. “The Impact of Intensive Care Strain on Patient Outcomes: A Multinational Observational Cohort (UNITE‐COVID) Study.” Critical Care 29: 329. 10.1186/s13054-025-05521-5. 40722093 PMC12306109

[jan70421-bib-0027] Koopman, W. J. , C. J. Watling , and K. A. LaDonna . 2020. “Autoethnography as a Strategy for Engaging in Reflexivity.” Global Qualitative Nursing Research 7: 1–9. 10.1177/2333393620970508. PMC7683839 33283020

[jan70421-bib-0028] Kurtzman, E. T. , L. V. Ghazal , S. Girouard , et al. 2022. “Nursing Workforce Challenges in the Postpandemic World.” Journal of Nursing Regulation 13, no. 2: 49–60. 10.1016/S2155-8256(22)00061-8. 35880143 PMC9299514

[jan70421-bib-0029] Leaver, C. A. , J. M. Stanley , and T. G. Veenema . 2022. “Impact of the COVID‐19 Pandemic on the Future of Nursing Education.” Academic Medicine 97, no. 3 Suppl: S82–S89. 10.1097/ACM.0000000000004528. 34789661 PMC8855777

[jan70421-bib-0030] Levecque, K. , F. Anseel , A. De Beuckelaer , J. Van der Heyden , and L. Gisle . 2017. “Work Organization and Mental Health Problems in PhD Students.” Research Policy 46, no. 4: 868–879. 10.1016/j.respol.2017.02.008.

[jan70421-bib-0031] Lopes, J. , V. Ranieri , T. Lambert , et al. 2017. “The Clinical Academic Workforce of the Future: A Cross‐Sectional Study of Factors Influencing Career Decision‐Making Among Clinical PhD Students at Two Research‐Intensive UK Universities.” BMJ Open 7: e016823. 10.1136/bmjopen-2017-016823. PMC5724086 28851792

[jan70421-bib-0032] Lyle, K. , S. Weller , G. Samuel , and A. M. Lucassen . 2023. “Beyond Regulatory Approaches to Ethics: Making Space for Ethical Preparedness in Healthcare Research.” Journal of Medical Ethics 49, no. 5: 352–356. 10.1136/medethics-2021-108102. 35725300 PMC10176337

[jan70421-bib-0033] Mart, M. F. , S. W. Roberson , B. Salas , P. P. Pandharipande , and E. W. Ely . 2021. “Prevention and Management of Delirium in the Intensive Care Unit.” Seminars in Respiratory and Critical Care Medicine 42, no. 1: 112–126. 10.1055/s-0040-1710572. 32746469 PMC7855536

[jan70421-bib-0034] Martínez‐García, I. , H. De Witte , J. García‐Martínez , and F. J. Cano‐García . 2024. “A Systematic Review and a Comprehensive Approach to PhD Students' Wellbeing.” Applied Psychology. Health and Well‐Being 16, no. 4: 1565–1583. 10.1111/aphw.12541. 38606943

[jan70421-bib-0035] McChesney, K. , J. Burford , and L. Frick . 2025. “Improving the Experiences of Doctoral Students at a Distance: Recommendations for Policy and Practice.” Perspectives: Policy and Practice in Higher Education: 1–8. 10.1080/13603108.2025.2506473.

[jan70421-bib-0036] Mondisa, J.‐L. , B. W.‐L. Packard , and B. L. Montgomery . 2021. “Understanding What STEM Mentoring Ecosystems Need to Thrive: A STEM‐ME Framework.” Mentoring & Tutoring: Partnership in Learning 29, no. 1: 110–135. 10.1080/13611267.2021.1899588.

[jan70421-bib-0037] Nästesjö, J. 2023. “Managing the Rules of Recognition: How Early Career Academics Negotiate Career Scripts Through Identity Work.” Studies in Higher Education 48, no. 4: 657–669. 10.1080/03075079.2022.2160974.

[jan70421-bib-0038] Nikbakht Nasrabadi, A. , S. Abbasi , A. Mardani , M. Maleki , and Z. Vlaisavljevic . 2022. “Experiences of Intensive Care Unit Nurses Working With COVID‐19 Patients: A Systematic Review and Meta‐Synthesis of Qualitative Studies.” Frontiers in Public Health 10: 1034624. 10.3389/fpubh.2022.1034624. 36466502 PMC9710282

[jan70421-bib-0039] Nowell, L. , T. Risling , S. Davidson , and K. King‐Shier . 2025. “An Innovative Doctor of Nursing Programme: Transforming Learning, Leadership and Health Systems.” Journal of Advanced Nursing 0: 1–11. 10.1111/jan.70143. PMC13069187 40811570

[jan70421-bib-0040] Pizzolato, D. , and K. Dierickx . 2022. “Reverse Mentoring to Enhance Research Integrity Climate.” BMC Research Notes 15: 209. 10.1186/s13104-022-06098-w. 35715865 PMC9205068

[jan70421-bib-0041] Qin, M. , Y. Gao , S. Guo , X. Lu , H. Zhu , and Y. Li . 2022. “Family Intervention for Delirium for Patients in the Intensive Care Unit: A Systematic Meta‐Analysis.” Journal of Clinical Neuroscience 96: 114–119. 10.1016/j.jocn.2021.11.011. 34838428

[jan70421-bib-0042] Samra, R. 2025. “Conceptualising Lived Experience in Mental Health Research: Problems, Insights and Implications.” Sociology of Health & Illness 47: e70039. 10.1111/1467-9566.70039. 40305649 PMC12043252

[jan70421-bib-0043] Sengul, T. , S. Sarikose , V. Lopez , and H. Kirkland‐Kyhn . 2025. “The Impact of Doctor of Nursing Practice Education on Career Advancement and Professional Satisfaction: A Scoping Review.” Journal of Advanced Nursing 0: 1–26. 10.1111/jan.16976. PMC12810671 40237610

[jan70421-bib-0044] Stubb, J. , K. Pyhältö , and K. Lonka . 2011. “Balancing Between Inspiration and Exhaustion: PhD Students' Experienced Socio‐Psychological Well‐Being.” Studies in Continuing Education 33, no. 1: 33–50. 10.1080/0158037X.2010.515572.

[jan70421-bib-0045] Sverdlik, A. , N. C. Hall , L. McAlpine , and K. Hubbard . 2018. “The PhD Experience: A Review of the Factors Influencing Doctoral Students' Completion, Achievement, and Well‐Being.” International Journal of Doctoral Studies 13: 361–388. 10.28945/4113.

[jan70421-bib-0046] Trusson, D. , E. Rowley , and L. Bramley . 2019. “A Mixed‐Methods Study of Challenges and Benefits of Clinical Academic Careers for Nurses, Midwives and Allied Health Professionals.” BMJ Open 9: e030595. 10.1136/bmjopen-2019-030595. PMC6797317 31594886

[jan70421-bib-0047] Tu, A. K. , J. R. Haney , K. O'Neill , et al. 2023. “Post‐Traumatic Growth in PhD Students During the COVID‐19 Pandemic.” Psychiatry Research Communications 3: 100104. 10.1016/j.psycom.2023.100104. 36743383 PMC9886426

[jan70421-bib-0048] van Dongen, L. J. C. , and T. B. Hafsteinsdóttir . 2022. “Leadership of PhD‐Prepared Nurses Working in Hospitals and Its Influence on Career Development: A Qualitative Study.” Journal of Clinical Nursing 31, no. 21–22: 3414–3427. 10.1111/jocn.16168. 34897871 PMC9787967

[jan70421-bib-0049] Wall, S. 2016. “Toward a Moderate Autoethnography.” International Journal of Qualitative Methods 15, no. 1: 1–9. 10.1177/1609406916674966.

[jan70421-bib-0050] Wu, C.‐J. , S.‐M. Chen , and M.‐A. Ramis . 2025. “Educational Challenges for Post‐Graduate Nursing Students Throughout the COVID‐19 Pandemic: A Scoping Review.” Nursing & Health Sciences 27: e70032. 10.1111/nhs.70032. 39821440 PMC11737895

[jan70421-bib-0051] Wu, X. 2022. “Tears and Cheers: A Narrative Inquiry of a Doctoral Student's Resilience in Study Abroad.” Frontiers in Psychology 13: 1071674. 10.3389/fpsyg.2022.1071674. 36582317 PMC9793775

[jan70421-bib-0052] Xia, L. , Z. Cao , and Y. Zhao . 2024. “Paradigm Transformation of Global Health Data Regulation: Challenges in Governance and Human Rights Protection of Cross‐Border Data Flows.” Risk Management and Healthcare Policy 17: 3291–3304. 10.2147/RMHP.S450082. 39720184 PMC11668341

[jan70421-bib-0053] Yang, Y. , and J. Cai . 2022. “Profiles of PhD Students' Satisfaction and Their Relationships With Demographic Characteristics and Academic Career Enthusiasm.” Frontiers in Psychology 13: 968541. 10.3389/fpsyg.2022.968541. 36389606 PMC9650635

[jan70421-bib-0054] Zhao, J.‐l. , F. Chen , and X.‐m. Jia . 2021. “The Development and Validation of the Doctoral Student Identity Scale.” Frontiers in Psychology 12: 688948. 10.3389/fpsyg.2021.688948. 34950078 PMC8689975

